# Engaging local youths in humanitarian response is not a matter of *if* but *how*

**DOI:** 10.1186/s41018-022-00118-x

**Published:** 2022-04-02

**Authors:** Abigael Apollo, Marcellus Forh Mbah

**Affiliations:** 1 Department of International Development, School of Arts and Humanity, Nottingham Trent University, Nottingham, UK; 2 Institute of Education, School of Social Sciences, Nottingham Trent University, Nottingham, UK

**Keywords:** Local youths, Humanitarian actors, Humanitarian/crises/emergency response

## Abstract

Despite being critical responders in humanitarian crises, local youths are continually left out of the humanitarian action agenda. This paper used a qualitative methodology to investigate local youths’ role in humanitarian response and their impacts and assessed how humanitarian actors influence the effectiveness of youth engagement. The data was collected through semi-structured interviews with local youths who participated in the Ebola response in Sierra Leone. Findings showed that young people are significantly contributing to crises response. However, they lack an enabling environment and support system to convert their skills into valuable humanitarian resources efficiently. Therefore, despite the rhetoric that many reports and policies reflect, this study establishes that the realities of youth engagement in humanitarian activities are often misunderstood and controlled for the self-interest of different actors other than youths themselves. It advocates for a renewed focus and support for young people’s skills as  paramount for effective humanitarian response and building back resilient communities after emergencies. Besides, engaging local youths in tackling crises empowers them with transferable skills and stimulates their passion for participating in development issues within their communities.

## Introduction

Engaging local youths in humanitarian response is not a matter of *if* but *how*. By examining local youths’ role in the Ebola response in Sierra Leone, this paper critically analyses local youth-led and youth-centred humanitarian responses.

The term ‘youth’ is a fluid concept with no fixed definition. For instance, the UN defines youth as persons aged 12 to 24 years (UNDESA 2019), but in some countries, this age ranges up to 35. The idea of youth can also be associated with a certain status in a society like having children, being married or owning property. However, for this paper, youths refer to persons aged between 15 and 35 years (African Union Commission 2006). While the term ‘local actors’ in humanitarian action remains undertheorized, subjective and applied variously with different organisations (Enria 2020), local youth actors here are contextually defined as young people who were vulnerable and were experiencing the crisis because they lived in the geographical location and had to live  with the consequences personally (Roepstorff 2020). While the vulnerability of these youths could  also have been varyingly influenced by multiple factors, including their age, economic status, education levels, and gender (Ruiz-Casares et al. (2017)), their proximity to or within the  crisis situation (location) is the critical defining factor in this study. Contrarily, humanitarian actors here refer to entities or experts, often affiliated with international organisations who chose to be involved in the Ebola response but may have had nothing personally to lose ((Roepstorff 2020). They may include national and international non-governmental organisations, foreign governments, donors, multilateral organisations and United Nations agencies (Spiegel 2017). In this paper, the Sierra Leone national government, which received funding to respond to Ebola, is also classified as a humanitarian actor. On the other hand, local youths are not affiliated with any entities but voluntarily and individually chose to participate in the crisis response.

Enria (2020) and Barnett and Walker (2015) argue that international humanitarian actors have long dominated humanitarian response, with top-down relief efforts. Indeed, local actors’ involvement in the humanitarian system is a current debate that could cause significant shifts in response activities (Robinson 2018). While local youths are often first responders when emergencies occur, Haynes and Tanner (2015) assert that humanitarian actors tend to treat them as passive victims with limited roles to play to communicate and respond to crises. The authors further emphasise that humanitarian organisations act as instructors to youth rather than key partners in response activities (ibid). Ruiz-Casares et al. (2017) also point out that humanitarian actors tend to overlook young people’s potential as capable agents during crises; instead, their vulnerability is emphasised. Yet, young people are the largest world population. UNDESA (2019) reports that 1.2 billion people worldwide are youth aged between 12 and 24 years, and the population is projected to increase to 1.4 billion by 2065. The largest increase is expected in sub-Saharan Africa, where most youths live in fragile and conflict-affected settings. Hence, the labelling and exclusion in humanitarian response further compound their already complex challenges during emergencies.

At the same time, humanitarian emergencies are increasing in frequency and severity globally, causing long-term impacts on all countries’ sustainable development (Barnett and Walker 2015; Jaff 2020). According to the United Nations’ (2018) report on global humanitarian trends, large-scale crises that required an internationally led response doubled from 2005 to 2017. From the ongoing wars in Afghanistan, Somalia, Yemen to the earthquakes in Asia, the frequent floods and disease outbreaks in Africa and the deadly coronavirus (Mbah et al. 2021), these humanitarian emergencies pose one of the most severe challenges to reducing poverty and existing inequalities in the world. Several studies (Barnett and Walker 2015; Burkle et al. 2014; Jaff 2020) also indicate that the number of people in need and targeted for assistance has increased recently. Furthermore, climate change worsens the situation as it causes disasters and heightened conflicts as populations fight for scarce resources (Apollo and Mbah 2021). Hence, humanitarian actors should partner with youths to sustainably mitigate, respond to and build back better communities resilient to the ever-increasing humanitarian emergencies. According to Gul (2020,2020), young people bring energy, creativity, motivation, capacity for mobilisation and technological know-how in emergency response.

This paper, therefore, examines the agency and capacity of local youths to engage in humanitarian crises, the barriers they face, including the identities humanitarian actors assign them, the lack of their voice representation in crisis response activities and how that ultimately influences the effectiveness of their performance. The research presented here is prompted by anecdotal field reports from development organisations which suggest that local youths are already making significant contributions in humanitarian response. Through this lens, and using data derived from in-depth semi-structured interviews with young people in Sierra Leone who participated in the Ebola response, the paper explores three linked areas of local youth engagement in humanitarian response. First, it investigates the current role of local youths in the humanitarian response space. Second, it assesses the barriers that hinder effective local youths’ engagement in humanitarian response. Third, it proposes strategies to engage local youths in humanitarian response meaningfully.

To satisfy the research objectives of this study, the following questions will be answered:

- In what ways are local youths engaging in humanitarian response?
- What barriers hinder effective local youths’ engagement in humanitarian response?
- What are the strategies to meaningfully engage local youth efforts in humanitarian response?

The structure of the remainder of this paper is as follows: The next section briefly discusses the history of young people in Sierra Leone and the Ebola outbreak in the West African country. The information sets out the lived realities of Young Sierra Leoneans and how Ebola impacted their experiences and role in the emergency response. The subsequent section provides an extensive review of the literature on the topic. Details on the methodology used to conduct the study are later captured, as well as findings from the empirical data. The concluding sections discuss the research results based on the thematic analysis of interview data and advance recommendations.

### Contextual background

It is essential to understand the history of young people in Sierra Leone throughout the civil war in the country. The historical context teaches us how young Sierra Leoneans have been discursively constructed and how such identities influence how they are engaged in humanitarian action. Further, the history also highlights the challenges that local youths face and how that shapes their social activism and, eventually, local development. This section also discusses the Ebola outbreak in the West African country and how the events impacted local youth’s role in the crisis response.

#### History of the youth population in Sierra Leone

For 11 years between 1991 and 2002, most youths in Sierra Leone lived through a civil war that left 70,000 casualties and 2.6 million people displaced (Kaldor and Vincent 2006). Years later, Sierra Leone was listed as one of the poorest countries (UNDP n.d.). Accordingly, the war has always been characterised as the ‘crisis of youth’ where social marginalisation and a desperate search for employment prompted many young Sierra Leoneans to embrace conflict and join Revolutionary United Front (RUF) insurgents (Fanthorpe and Maconachie 2010). The youth’s fundamental imperative for self-empowerment, the authors argue, motivated them to join any process that in their worldview would improve their living conditions (ibid).

Efevbera and Betancourt (2016) report that many youths were used politically during the war, only to be marginalised later during the peace process. As a result, their problems were neither represented nor heard. McIntyre and Thusi (2003) also assert that while the UN rightly reported Sierra Leone’s peace process as successful, it failed to resolve the many social, political and economic issues that motivated youths to join the fight initially. Additionally, the lived realities of many youths who were not involved in the fighting, although significantly affected by the war, remained understudied (Peters 2011). Such alienation, therefore, posed more complex challenges for the country’s youthful population, including a collapsed education system, high levels of unemployment and corruption, compounded with uncertain futures (Efevbera and Betancourt 2016).

The post-war period between 2002 and 2005 led to a remarkable upsurge of young people in self-organised social activism to hopefully empower themselves economically and enhance local development. However, Efevbera and Betancourt (2016) point out that the lack of jobs in the diamond mining sector, which holds historical importance and is a useful means of income for young, single, uneducated and unemployed Sierra Leoneans, was disrupted. The industry generated intensified politics between many young ex-combatants who migrated for mining labour jobs and ruling chiefs in Kono, the largest mining district in Sierra Leone (Fanthorpe and Maconachie 2010). The authors posit that the migrant rush into the region was of great concern to the locals who felt the influx of strangers supported illicit mining and stole their rightful wealth. The chiefs also felt that the youth had become selfish and took advantage of the available community resources (ibid). Yet, for many young people, mining represented an opportunity to escape rural poverty and injustice.

While young Sierra Leoneans formed advocacy groups to control opportunistic diamond digging and smuggling, the youth groups soon became the target for political competitions that resumed after the war. Fanthorpe and Maconachie (2010) report that many youth leaders forged new careers in party politics and left their mandate of mobilising youths for good causes calling out the injustices in diamond governance. Consequently, youth unemployment increased in Kono district and wider Sierra Leone as supporters drifted away and mining shifted to capital-intensive modes of extraction requiring less unskilled labour. For these reasons, young Sierra Leoneans remained with little economic bargaining power and have continued to live in conditions of extreme poverty.

#### The Ebola outbreak in Sierra Leone

The Ebola virus disease (EVD) outbreak began in December 2013 in West Africa, spreading from Guinea, Liberia and Sierra Leone (Centre for Disease Control and Prevention (CDC) 2019). With rapid increasing infection rates, the World Health Organization declared a public health emergency of international concern in July 2014 (Enria 2020). By September 2014, the West Africa Ebola outbreak was described as one of the largest in history since the disease’s discovery in 1976 (World Health Organization 2015). Ebola-affected some of the most vulnerable communities and countries still recovering from decades of destructive civil war and unrest. According to the Centre for Disease Control and Prevention (CDC) (2019), Ebola caused trendemous negative impacts with 11,300 deaths, 28,000 confirmed cases, 3.5 billion USD spent on the response activities and economic losses costing 2 billion USD. Moreover, an initial underestimation of the outbreak scope caused delays in funding and a slow start to the response.

Sierra Leone documented the highest cases among the affected countries, with 14,124 infections and 3955 deaths (World Health Organization 2015). The pandemic reversed the development progress that the country had achieved since the decade of war. These challenges notwithstanding, Farrar (2019) reports that local youths in Sierra Leone teamed up to contain the disease. Many NGOs chose to close operations, leaving the burden for fighting the epidemic with local responders. As the elderly also stepped back, fearing for their health, young Sierra Leoneans proved capable agents in humanitarian response as they helped their communities fight the deadly virus (Plan International 2016).

### Literature review

The literature will be examined under three categories, namely youth participation in humanitarian action, power distribution and politics in humanitarian action and categorisation of local youths in humanitarian actions.

#### Youth participation in humanitarian action

Chauke (2020), Bocking-Welch (2016) and Efevbera and Betancourt (2016) observe that young people are keen to lead crises response initiatives and support their communities towards recovery. From creating awareness, distributing food, accelerating evacuation and mobilising resources (Haynes and Tanner 2015), youths are uniquely placed to play a valuable role in locally led humanitarian actions using their networks across their communities and great innovative approaches. The context of the novel global corona pandemic serves as an example of how young people are already immensely participating in humanitarian response. For instance, in South Africa, local youths in Cape Town helped spread accurate information on safety measures through social media platforms as the country observed social distancing (Chauke 2020). In Kenya, youths in the urban slums initiated food banks to distribute food to vulnerable populations who lost their jobs in the informal sector (OECD 2020). All these undertakings and many others exemplify how young people’s work can positively shape the humanitarian response.

Despite the contributions mentioned above, the idea of youth participation and what it means in practice remains contested. Haynes and Tanner (2015) observe that most emergency response activities are not youth-led nor youth-centred. Drawing from Gul’s (2020) study, youth-led actions do not eliminate the expert’s role; instead, it is a specific relationship where humanitarian actors support local youths to gain skills, information and capacity to make decisions during humanitarian action. Maximising the role of local youths entails supporting them to design action plans and allowing them to lead the implementation process, which ensures continuity in response (Welch 2016). Besides, previous studies suggest that engaging local youths in humanitarian response and recovery can reduce the cost and the need for international humanitarian support, improve humanitarian effectiveness and strengthen communities’ resilience (Chauke 2020; Mitchell et al. 2008).

Sociologist Roger Hart’s ladder of young people’s participation is an effective tool to evaluate the level of meaningful youth participation in humanitarian response. As shown in Table 1 below, the ladder has different rungs that depict different degrees of involvement. According to Hart (1992), the bottom three rungs (manipulation, decoration and tokenism) show no participation where young people have no agency in the projects/activities. The following middle three rungs show moderate participation where youths are well informed and consulted about the activities, but they still lack access to leading decision-making. The last two rungs indicate full participation with young people initiating ideas and leading decision-making and implementation processes. However, Ramey et al. (2017) contest that lower rungs of Hart’s ladder are not intrinsically worse than the higher levels, especially in circumstances where young people may need adults’ support and guidance to fully participate in leadership. Therefore, this paper advocates for partnerships that constitute youth-led ideas and shared decision-making with humanitarian actors to provide opportunities for learning from each other.

**Table 1:**
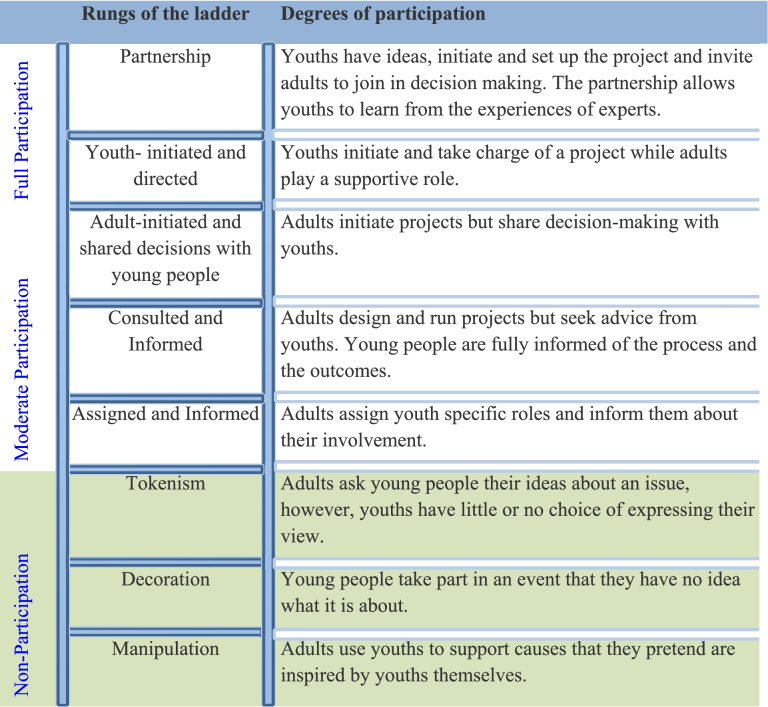
Ladder of participation adapted from Hart 1992

In a humanitarian work context, local youths may help in advocacy; however, it does not become actual participation until their views are translated into action through planning activities. As a case in point, most of the sections in the ladder involve some elements of manipulation of youth to achieve results that exclude their consultations or awareness about the issue at hand. Indeed, this practice seems to be a lived reality of many youths who engage in humanitarian activities.

This section provides the theory through which the paper will analyse youth decision-making during humanitarian response. The findings based on Hart’s concept will assess whether youth voices were truly represented as first responders and crucial humanitarian partners.

#### Power distribution and politics in humanitarian action

While the humanitarian system has expanded over time, analyses suggest that transformative change that meaningfully engages all actors, including local youths, is rare (Spiegel 2017; Hilhorst 2018; Bah 2013; Barnett and Walker 2015). Hilhorst (2018) argues that the humanitarian system’s fundamental power structures, institutions, and architecture have remained remarkably similar over the years. The current system, Hilhorst maintains, has long been dominated by classical paradigms centred on big institutions who use their advantages to organise emergency response systems that preserve their power. The author further describes the humanitarian field as ‘an arena where actors use social negotiations and power to shape the everyday practices of policy and implementation based on their understanding, strategies, ambition and assumptions they have about the local context and the people they serve’ (Hilhorst 2018 p. 1120). Echoing the arguments of Hilhorst (2018), Barnett and Walker (2015) refer to this tight-knit group of elites as the ‘humanitarian club.’ According to the authors, this club holds real power over how the world responds to emergencies by setting the rules. It has the ability to push the reforms that serve its interest and deter measures that threaten its privileges. Even though this ‘club’ deserves credit for the successful reforms like championing for flexible humanitarian financing, Konyndyk and Worden (2019) contend, it is also responsible for the lack of meaningful systematic change and engagement of local youths in humanitarian action.

Pacitto and Fiddian-Qasmiyeh (2013) assert that these external humanitarian actors cannot understand local populations’ lived realities in crises, including young people, women, the elderly and thus overlook some of their most pressing challenges. Roepstorff (2020) argues that even though the importance of local actors has been acknowledged in the UN resolution of 1991 and the Red Cross code of conduct of 1994, recent evaluations have revealed a lack of implementation in practice. Certainly, crisis response remains something done to affected populations, not alongside them (Barnett and Walker 2015). Haynes and Tanner (2015) report that young people are continuously treated with mistrust and preconceived notions that they need capacity building to respond to ‘big’ emergencies (Ibid). While Spiegel (2017) agrees with the idea that international organisations have a better capacity, including a robust command and control structure to lead complex humanitarian emergencies, Hilhorst (2018) labels the concept of ‘capacity building’ as a terrible term that conveys a non-agentive framework built up by external forces. According to the author, the discourse of capacity building for local actors is pervasive and is accepted even in areas where international humanitarian operations have been ongoing for long periods. In this context, the rhetoric always seems to depict what local youth responders are missing regarding educational qualification rather than emphasising their strengths. Thus, such discourse reinforces the existing power relations in the process and misses out on the possibility of mutuality in humanitarian response (Roepstorff 2020).

Gul (2020) also points out that involving young people in decision-making during humanitarian response is often stated but rarely acted upon in policy development. For instance, the UN developed the Compact for Young People in Humanitarian Action (CYPHA), launched during the World Humanitarian Summit of 2016, emphasising the urgent need to engage young people in humanitarian response activities and act as a deliberate step for implementation (UNFPA 2018). The compact seeks to engage young people in five key areas of focus in emergencies; service delivery, data and knowledge, resources, participation and local action. A year before, the United Nations security council had adopted a resolution on youth, peace and security, which underscores the role of youth in conflict prevention and building and maintaining peace (United Nations 2015). While these commitments have played a critical role in recognising youths as agents of change in crises, they are yet to operationalise in the field (Gul 2020). Spiegel (2017) ascertains that resolutions are essential to provide the basis for action, but they only remain words on paper without substantial consequences of breaking them. Indeed, that seems to be the practice when involving local youths in emergency response.

As Bah also rightly puts, ‘the criticism on humanitarian intervention is not based on its moral imperative to protect the vulnerable populations but rather, on its sovereignty and reinforcement of colonial vestiges’ (Bah 2013 p7). This sovereignty, Bah notes, assigns developed countries and Western-led organisations’ superiority over vulnerable populations (Roepstorff 2020). Pacitto and Fiddian-Qasmiyeh (2013) write that this dominance is further promoted by the labelling of humanitarian response as a ‘Western creation’ by academics and practitioners, thus undermining the significant efforts of local responders during crises. For instance, local youths’ efforts as the first responders during the Ebola response gained little public attention while all the credit was given to a set of well-known and established humanitarian actors (Christensen et al. 2020). According to Roepstorff (2020), such control practices create suspicion and ultimately the rejection of humanitarian action when local people view them as a continuation of colonisation. Bah (2013) maintains; even though humanitarian governance is driven by the ethos of helping the most vulnerable, in doing so, it should not involve practices that rule or exclude the very people it is trying to help. The idea is not for external humanitarian actors to cede power to local youths but rather to involve them as essential partners in humanitarian response (Haynes and Tanner 2015). The integration of the local youths should not be based on relationships or politics but primarily on humanitarian needs.

Indeed, humanitarians have grown aware of their faults and have made remarkable progress in improving competence, coordination and professionalism in humanitarian actions (Barnett and Walker 2015). However, these gains have not been matched with equal progress in localising humanitarian response. Many partnerships with local youths remain reactive, influenced by ad hoc interactions at the point of crisis occurrence (Mitchell et al. 2008). The author maintains that at this point, it is already too late, and young people cannot meaningfully lead humanitarian responses in their communities. Instead, organisations should involve young people in designing response plans and consult them in all the stages of disaster response from prevention, mitigation and preparation. That way, local youths can be equipped with adequate skills combined with their creative abilities to act quickly when crises hit and further build back resilient communities.

Based on the discussion above, fundamental analysis and critical engagement with the dominant practices and underlying assumptions motivate this study to avoid reproducing problematic exclusionary humanitarian practices. The inequalities designate a need to challenge the negative power dynamics in the current humanitarian system and make it more accessible to local youths as key actors.

#### Categorisation of local youth in humanitarian action

Understanding how critical humanitarian agencies construct youth identities is essential as such framings significantly shape their humanitarian response and international development engagements. Haynes and Tanner (2015) confirm this argument and acknowledge that the problem of defining youths during crises has had severe implications for policy formulation and, ultimately, programme design. It is undeniable that categorising people is a crucial aspect of humanitarian response. It influences agencies’ eligibility factors for service provision, during which they constantly have to decide which groups to include and exclude (Hilhorst 2018). However, labelling goes further and can have tremendous effects (Haynes and Tanner 2015; Mcintyre and Thusi 2003). The labelled, and in this case, local youths, become defined as the object of the labelling subject—the humanitarian actors. As Gul (2020) also emphasises, influential humanitarian agencies have the power to construct youth identities and have the power to act on those framings and develop structures that reinforce the very identities they have constructed. Consequently, young people are traditionally trapped in the dichotomous paradigm of being portrayed as ‘troublesome population that needs to be controlled,’ ‘perceived to be vulnerable and needs protection’ or ‘talented resources that need to be harnessed’ (Gul 2020; Ruiz-Casares et al. 2017; Haynes and Tanner 2015).

##### Youths perceived to be vulnerable and in need of protection

The construction of youths in crises as victims is the most dominant. For instance, several studies have been conducted on the negative impacts of war on young people in Sierra Leone (Betancourt et al. 2014; Fanthorpe and Maconachie 2010; Efevbera and Betancourt 2016). Youths are also discursively constructed as victims in the policy frameworks and reports of the United Nations. Specifically, the UN Compact for Young People in Humanitarian Action (CYPHA) states that ‘young people displaced find themselves in situations they have no control over’. ‘They are forced to leave their homes due to conflict and flee the effects of climate change’ (UNFPA 2018 p.42). Further, while defining youths in armed conflict, the UN’s World Programme of Action for Youth describes them as ‘main victims’ who are ‘abducted, traumatised, murdered, forcibly displaced and left as orphans’ (United Nations 2010, p. 55).

Whereas this vulnerability approach galvanises attention and resources to young people during a crisis, debates are rising whether there is an overemphasis on youth development challenges at the expense of their strengths and capabilities (Mcintyre and Thusi 2003; Kemper 2007; Haynes and Tanner 2015; Gul 2020). In the above instances, the UN differentiates youths from ‘others’ who have the power to violate their rights, thus emphasising the dominant narrative of youths requiring protection rather than active participants in humanitarian activities. Kemper (2007) asserts that such discourses entrench the stereotypes of victimhood and passivity, which are then accepted as ‘truths.’ Gul (2020) also rejects the overprotection of young people as it often leads to their exclusion from critical processes that affect them and eventually substantial loss for the communities they live. Mitchell, Tanner and Haynes (2009) also observe that many organisations find it challenging to design and sustain interventions that allow young people to participate on an equal level as adults due to victimhood labels, not considering that many youths in developing countries take up adult responsibilities as part of their daily lives.

##### Youth as ‘agitators’ that need to be controlled

The construction of youth as agents of conflict has achieved dominance over the years. Alfy (2016), Rahman (2014) and Urdal (2004) link the increasing population of young people referred to as the ‘youth bulge’ to increased conflicts which often develop into long-term humanitarian crises. According to Rahman (2014), societies with high youth populations are characterised by rampant unemployment rates, with idle youth susceptible to radical group recruitments. Contrarily, according to Urdal (2004), well-educated youth have better income opportunities and more to lose; thus, they would be less interested in causing conflict. However, in its conclusion, the youth bulge analysis ignores most youth who avoid war (Peters 2011). Neither does it account for the experiences of most youths who are keen to promote peacebuilding but have continuously been sidelined in conflict resolution. For example, McIntyre and Thusi (2003) observe that young people were excluded from peace negotiation processes after the war in Sierra Leone.

Similarly, the United Nations represents youth with child-like character, lacking the skills to manage their emotions in non-violent ways. It reports a need to ‘teach young people to communicate without violence’ and ‘sensitise them about the detrimental effects of violence on their communities’ (United Nations 2010, p. 37). The UN typically situates young combatants in the African and Asian regions, where they ‘are poor, angry, frustrated, uneducated and lacking economic opportunities’ (Ibid p. 78). By contrast, youths from the global north are represented as rational, with good economic stability; thus, they do not need to revolt violently. Such orientalism promotes the biassed discourses of the uncontrollable youth population in Africa, the Middle East and Latin America, all considered hotspots.

Hence, in positioning young people as ‘troublemakers,’ the UN creates its own identity as the leading force for young people during humanitarian crises. Their emphasis on problematic youth creates the need for command structures to control the ‘uneducated immature’ youth, who, if not led, can cause further detrimental impacts during an emergency response. Such constructions, therefore, hinder meaningful engagement in conflict-related crises, as young people are viewed as the very cause of the problem at hand.

##### Youth as ‘exuberant human resources’ that need to be harnessed

Gul (2020) writes that there has been a shift in the global policy rhetoric of controlling the ‘problematic’ youth to empowerment through civic engagements in the last decade. In its Compact for Young People in Humanitarian Action (CYPHA), the UN acknowledges that ‘the collective energy and dynamism of the world’s largest population of youths is powerful to respond to the ever-increasing disasters’ (UNFPA 2018 p5). While the UN rightfully recognises youth as capable agents in crises, Gul (2020) reports that implementing these policies in the field remains a challenge as the traditional negative perceptions of youth persist in the humanitarian sector. Further, even though the intentions are right, the author argues that the constructions represent the large youth population as the ‘newly discovered’ reserve of ‘human capital for humanitarian agencies’. According to McIntyre and Thusi (2003), such inclusion of local youths in humanitarian response is often intended to give legitimacy to belligerent parties rather than partnering with them as a rightful responsibility.

Considering the above three categorisations of young people, it is evident that youths’ representation significantly shapes youth-centred humanitarian response. As Androutsopoulos and Georgakopoulou (2008) suggest, any discursive construction has an underlying strategy, whether conscious or unconscious. Consequently, influential humanitarian actors seem to assign young people the identities that suit their organisational plans in a crisis setting. Therefore, understanding the construction of youth identity they accept is vital for this study to assess how it impacts their approaches and engagement with local youths during humanitarian response. Besides, such labelling also influences how local youths internalise their identities and, ultimately, their role in emergencies.

The above discussions show that local youths have the capacity and agency to engage in humanitarian response. However, unequal power distributions and the identities humanitarian actors assign seemingly hinder their effective engagement in responding to crises and supporting their communities to recover from emergencies. Therefore, within this gap, there is an opportunity to explore in detail the nature of local youths’ engagement in crisis response, including the barriers they face and their perceptions of risk and their roles in the humanitarian space. With the findings, we can target strategies to eliminate these barriers and support local youth efforts for effective humanitarian response. Such steps will ensure timely and cost-effective crisis response and promote sustainable recovery after emergencies. Meaningful local youth engagement would also empower young people with more skills to foster local development.

### Theoretical underpinning

This paper is grounded on Amartya Sen’s capability approach, a theoretical framework that the economist first introduced in 1985 for assessing well-being without imposing one’s notion of what a good life should contain (Grande 2014). The theory entails five main elements; capability, functioning, individual endowments, conversion factors and agency, which all correlate to influence an individual’s achieved outcomes of life (Chiappero and Roche 2009). While applications of the theory have so far ranged from gender inequality issues to education projects (Bhasin and Jain 2021; Hart 2012), no academic research has used the capability approach to understand local ‘youths’ engagement in humanitarian response.

As established in the literature review above, young people already have the personal endowments, including unique skill sets and local knowledge, to engage in humanitarian response. However, as Sen argues, it is not enough to have the resources like skills if there is no freedom to convert them to valuable functionings. Therefore, this paper adopts Sen’s theory to assess the opportunities that in reality exist for local youths to participate in humanitarian response and how conversion factors like power structures within humanitarian institutions shape young people’s ability to turn these skills into their desired being and doings in crisis settings. The theory is essential for this study as it does not impose ideas but instead advocates for empowerment via participatory development, which encompasses affected populations sharing control over decisions and resources (Robeyns 2005). It recognises the crucial role of institutional agency in promoting capabilities over time to achieve the desired change. In this case, the capability approach recommends that humanitarian actors create opportunities for youths’ equal participation in decision-making during crises response. Hence, this theory will provide a more coherent assessment that advocates for converting young people’s skills into valuable human resources and the required means to support youths to fully realise their capability’s in humanitarian response.

## Methodology

This study is guided by an interpretivist paradigm and utilises a qualitative research methodology and in-depth key informant interviewing method. Scotland (2012) argues that an interpretivist paradigm is well suited to explore complex social processes, where quantitative evidence may be biassed or inaccurate. Hence, the approach effectively explored how expert humanitarian actors relate and work with local youths in multiplex emergency settings. Additionally, a qualitative design was the most appropriate for this study. It allowed the researcher to collect and present data from the perspective of the subjective realities of local youths involved in humanitarian response and the meanings they attach to these realities (Choy 2014). Harding (2018) further points out; the qualitative research method goes beyond surface appearance to gain thick descriptions of the various factors about the topic of study. Therefore, through in-depth semi-structured interviews, the participants were allowed to play a more active role, expressing their opinions, values and priorities in an open-ended inquiry, thus raising broader issues. The approach also challenged some of this study’s assumptions (Choy 2014) and raised significant issues concerning local youth engagement in crises.

While this study assembled narratives and meanings from the interviews, it also recognised the respondents’ and the interviewer’s active subjectivity and positions. Davies and Harré (1990) describe positioning as the discursive practice whereby people adopt their worldviews about a research task. Positionality influences how research is conducted, its outcomes and the results (ibid). For that reason, an inductive analysis method was adopted to avoid evident and systematic bias and let the data speak for itself. Care was also taken to ensure that interviews encompassed various perspectives and forms of young people’s engagement in humanitarian response.

### Sample selection criteria

Study participants were selected using the purposive then snowball sampling method. The criteria ensured that respondents were chosen for their role and experiential learning of the humanitarian response rather than their representativeness (Sharma 2017). We began by initiating contact with suitable respondent/s and then asking if they knew other potential participants for the project (Harding 2018). First, researchers contacted two key persons (youth leaders) who participated in the Ebola response in Sierra Leone. They were introduced to the study and asked for referrals among their acquittances. They were explained a need for participants of both gender and specifically local youths who volunteered or were temporarily employed by humanitarian organisations during the Ebola response. The youth leaders identified possible participants and shared their contacts. Through WhatsApp, each potential interviewee was individually engaged, explaining the reasons for the study and confirming that they met the selection criteria. The initial communication was helpful as it enabled a good rapport with the participants. After reconfirming their willingness to be interviewed, they were each sent a project form, a consent form and an online interview link.

While the geographical representativeness of the respondents was not relevant for this study, their experiences working in some of the worst-hit regions in Sierra Leone, Port Loko, Kono and Freetown represent the encounters of many young people who participated in the Ebola response. The youths played key roles in food distribution, advocacy, community mobilisation and sensitisation, conducting burial services for infected corpses, driving etc. The educational level of the respondents was average, with many having attained high school education as the highest level. Even though education level was not a mandatory selection criterion, it promoted the study’s reliability and validity, emphasising the significance of non-expert humanitarian actors. Indeed, this reflects local youth responders in crises, especially in ‘developing’ countries with high inequalities in seeking higher education. Nevertheless, these young people still play a critical role in emergency response.

### Interview guide

While an interview guide was developed based on this study’s objectives, it remained flexible to emerging themes during data collection. The questions centred on seven key sections; the personal experiences of local youths during the Ebola emergency, their role and motivation to participate in the response, willingness of humanitarian experts to engage local youths, work relations of youth with humanitarian actors, including the support and challenges they faced, youth engagement after the crisis, impacts of youth engagement on local development and recommendations for meaningful participation in the future. The development of the interview guide was guided by previous research into young people’s involvement in disaster management (Haynes and Tanner 2015; OECD 2020), a list of emerging issues in youth volunteering (Gul 2020) and the localisation agenda in humanitarian action (Roepstorff 2020).

### Data collection and analysis

Key informant interviews were conducted with six males and four females between June and July 2021. While this number does not capture a large group of local youth’s experiences in humanitarian response, we captured a detailed picture of participants’ realities that is often lost in a large-scale quantitative study. We also focused on getting a high representation of diverse roles that young people took up during the crisis response. In addition, forty percent of the respondents were team leaders, giving them more overview of the recurrent issues that local youth responders encountered during the Ebola response. As this research was not funded, interviews were conducted online via MS Teams and Zoom to cut the cost of travelling to Sierra Leon and observe lockdown safety measures during the COVID-19 pandemic. While Gabrium and Holstein (2012) contend that crucial informant interviews (KIIs) are limited on generalizability, they served as a means of co-production for this research. Participants took part voluntarily, having first provided a written informed consent form. While no payment was offered for their time, they were  reimbursed the Internet costs of their participation. The total number of interviews-10 was sufficient to enable critical analysis with clear recurring themes emerging. The data collected digitally was recorded on MS Teams.

After conducting the interviews, the recordings were transcribed word by word, eliminating only filler speech, resulting in 14,171 words of transcription. The transcription process was beneficial as it helped in the understanding of the data and tentatively pointed to useful information. The transcripts were later analysed thematically using both deductive and inductive methods. Joffe (2012) defines a deductive method as applying predetermined codes on the data, while an inductive process entails coding data based on emerging themes. Both methods were essential for this study; the deductive approach focused on the research objectives, while the inductive method promoted flexibility for relevant emerging themes (Alhojailan 2012). First, the transcripts were read repeatedly to ensure familiarisation with the data while taking preliminary notes. It was then followed by rereading the transcripts to identify commonalities and variances suitable for the research objectives, which were then coded with colours. During this process, the recurring themes were named with keywords. Finally, the main thematic categories were reviewed carefully and organised into specific findings, supported by the evidence of direct quotations.

### Ethical considerations

This study adhered to the ethical principles and standards of research. We sought signed informed consent forms from the participants and fully informed them about their ability to withdraw their consent at any point during data collection. As the interviews were conducted online, there was no physical severe health risk expected. The participants’ names were kept anonymous to ensure confidentiality and privacy, as Harding (2018) advocates. Respondents were also given opportunities to ask any questions about the research before and after the interviews. We stored the collected data in a password-secured electronic file, and we have only used it for the sole purposes of this research.

### Study limitations

The limitation of this study lay in the small sample size, which limited the generalisability of the results. The researchers' preconceived views may have also influenced the authenticity of the findings and directions of the study. Conducting interviews online could also have impacted the rapport developed between the researchers and the participants, affecting data depth. In addition, the perspectives of humanitarian actors working with young people during response activities were not captured in this study, thus potentially creating a one-sided narrative. However, conducting a thorough literature review and analysing all collected data curbed most of these challenges. While this work is empirically based on humanitarian response in one setting, the local youth engagement issues that emerged from it are to a large degree generic. Future research would benefit from engaging a wide range of young people who have participated in different contextual humanitarian emergencies.

## Findings

This part of the paper is divided into three main sections, representing the objectives of the study. Each section discusses various emerging subthemes informed by the findings of the research inquiries. The first section discusses young people’s perception of risk and the willingness of external actors to engage them in humanitarian response. The section also explores young people’s motivations to participate in Ebola response activities and the impacts of their involvement on the crisis response and local development. The second section discusses young peoples’ work-related challenges like the lack of involvement in decision-making involvement, age discrimination, low job ranking and compensation, and lack of engagement after crises. Lastly, the third section discusses the strategies, recommended by the youth, to meaningfully engage young people in humanitarian response.

### Role of local youths in the humanitarian response space

#### Youth's perception of their risk versus humanitarian actors’ perceptions of youths’ risk

It was essential to understand local youth’s perception of the risk they faced in humanitarian response and how it influenced their engagement. When asked their thoughts about the UN’s statement that young people are at the highest risk when a crisis occurs, more than half of the participants reported a different observation. According to the institution’s report where we drew the statement, the UN affirms youth vulnerability due to the challenges they face in crises, like missing education, increased sexual violence and job losses, among other issues (UNFPA 2018, p 11). While this standing is right, contrarily, the interviewees felt that youths were exposed to high risk because they were the frontline workers when an emergency occurred. Seventy percent of the participants asserted that organisations and governments always seek youth to be at the frontline of humanitarian response due to their high population and the belief that they have strong immunity.

Of course, even when it comes to taking the risk during Ebola, it was just young people who were being exposed to it. We were at the forefront. The old people were at the back pushing us to go and fight.

Another person emphasised that young people’s identity as the strong workforce is common with most emergencies, even with the COVID-19 response. However, when it comes to safety, she said, older people are given priority.

Even with the current Covid, they say, ‘you young people are strong, so allow old people to take the vaccine first.’ But when it comes to the fight, they say they need young people. Sometimes we are not given the protective gear while we are going to work.

The above responses distinctly show that these youths did not view themselves as vulnerable victims in crises due to their nature of being ‘young’, as is the common identity assigned by humanitarian actors. Instead, they felt their vulnerability was exacerbated by the lack of adequate protection as frontline workers during emergency response. Consequently, a few interviewees challenged the UN to change its mentality and emphasis on youth vulnerability as they were already immensely engaging in crisis response.

The first scenario in the UN report that emphasises young people’s victimhood is different from the testimonies of the participants about what in reality happens during crisis response. The youths in the latter case were identified as human resources, being encouraged to fight Ebola, and in extreme circumstances at the expense of their safety. Indeed, this reveals that youth identity in emergency settings can be constructed differently based on the agenda and the needs of different actors other than youths themselves, thus restraining young people from their agency in humanitarian response.

#### The willingness of external actors to engage local youths

Humanitarian actors’ construction of young people also influenced their willingness to engage local youths in the Ebola response. More than half of the interviewees felt that NGOs and the government were keen to work with local youths during the Ebola response as they had no options. As some respondents asserted, the older adults feared for their health and were reluctant to participate in the humanitarian response;

All organisations worked with young people because there was no option. They were willing. Like the burial team where I worked, 99% was comprised of young people.

Twenty percent of the participants observed that local NGOs were initially unwilling to engage young people during the first stages of the Ebola outbreak. Still, they later recognised the critical role of Young Sierra Leoneans in the crisis response when Ebola cases started increasing.

Initially there was an oversight then when they started seeing youths doing a good work, then they said, “ooh, they should involve young people”.

The above responses suggest that the willingness of humanitarian actors to engage local youths in the Ebola response was more inclined towards young people’s availability as human resources and not on their right as equal partners in emergency response. For those reasons, some NGOs felt reluctant to support local youth participation until they realised there were no other options. Perhaps, if there were options and there was no demand for handful young people to help, local youths would have been excluded from the whole process.

On the contrary, a section of the youths acknowledged that notable youth-led organisations worked well with local youths because they believed youths could perform and have the energy and capacity. Twenty percent expressed that international organisations came in and inspired young people to volunteer more because they brought safety materials for working. Ten percent observed that the external experts were very friendly and created an atmosphere to feel safe. One youth reinstated that ‘the international community, they play a big role. They come as a saviour’.

According to the testimony above, the international community which in this case refers to humanitarian actors brought protective gear that made young people to feel safe enough to want to participate in the Ebola response. Given the nature of the Ebola pandemic, the safety materials international organisations provided motivated young Sierra Leoneans to join the risky fight despite the discouragement that many reported from their families for fear of their health. Indeed, 10% of the respondents perceived that people working in the frontline observed more safety than people in the community. One further affirmed that ‘I believe working in the frontline will even make you safer’.

Apart from providing safety materials, more than half of the respondents confirmed that humanitarian organisations provided them with training on communication skills, first aid delivery, peer counselling and safeguarding to prepare them for work during the Ebola response. The training, therefore, instilled confidence in the youth volunteers as they had not engaged in a humanitarian response before. One youth remarked as follows:

The training, in fact, gave me a lot of motivation and strength to do the work in the field because I was prepared for it despite the challenges I faced.

Apart from the training, twenty percent of the interviewees also noted that NGOs linked them up with various community networks to conduct community sensitisation. These key contacts, including local chiefs and village elders, helped mobilise people for meetings.

#### Motivation to participate in humanitarian response

When the youth were asked how they came to participate in the Ebola response, altruism and the passion for protecting their communities emerged as the leading motivation to participate. Sixty percent of the respondents reported that they chose to volunteer due to the amount of suffering they saw in their community; hence, they felt the need to sacrifice their time and resources to support response activities.

I didn't think of money; I know the benefits when I take part. It makes me know that I contributed to changing lives, and when I see people I helped, it makes me feel that I am a good citizen, that I am a humanitarian.

Twenty percent of the participants revealed that they engaged in the Ebola response to advocate for young people’s protection, having suffered abuse before. Further, one youth expressed that they wanted ‘to challenge the stereotypes that people have that it is only adults who can make decisions because that did not work from their experience.’ Arguably, these personal experiences possibly made the youths’ participation in the response powerful and influential*.*

Apart from creating impact, forty percent of the interviewees highlighted that high youth unemployment motivated them to take up the available volunteer opportunities during the Ebola response to earn some stipend. Twenty percent of the respondents communicated that their peers persuaded them and linked them up with organisations for opportunities to volunteer. At the same time, ten percent expressed that they wanted to explore and use their skills during the emergency response. Table 2 summarises the motivations to participate from the highest to the lowest.

**Table 2:** Motivations for participation

Motivation for participation	Number of participants
Passion to protect their communities and make a difference	60%
Need for income	40%
Beliefs in the possibility of influencing decision making	20%
Influence of people around them	20%
Explore and learn new skills	10%

#### Impacts of local youth engagement in humanitarian response

When assessing the impacts of local youth engagement in the Ebola response, this study established that young people used their creativity and unique skill sets to solve some of the most pressing challenges during the Ebola outbreak.

Eighty percent of the interviewees noted that a lack of trust, many myths about the disease and conflicting information shared with the general public were the most significant drivers to Ebola’s spread. For instance, there was an indication from participants that many villagers believed that Ebola was caused by witchcraft. Moreover, some community members preferred seeking treatment from traditional healers who could not provide appropriate care for Ebola patients. Therefore, with their partnership with global medical experts, local youths played a crucial role in countering the negative perceptions by developing community-informed Ebola messages, which they communicated through two-way dialogues with community members. Considering the views and experiences of the affected population, as some interviewees reported, ensured that the disseminated messages were relevant and understood as intended. As a result, people began to observe the set Ebola safety measures. The following testimony summarises it:

We reduced the tendency of fear about Ebola. We changed the mindsets of people and created confidence among community members during the Ebola response. We also ensured that people had the correct information and also raised the concerns of the vulnerable people. We encouraged people to listen to radios.

The participants in this study also detailed that young Sierra Leoneans created by-laws to monitor populations’ behaviour to contain Ebola. Youth leaders set up checkpoints in every chiefdom through their youth groups with rigorous contact tracing and surveillance. Through these initiatives, as seventy percent of the interviewees expressed, encouraged people to increasingly report suspected Ebola cases to the health facilities, significantly reducing the disease’s spread.

Whoever had a visitor in their house, in their village, they had to report that to a committee that consisted of local youths. If someone is sick and the family hides, the youths would consider that as a crime. People who did not observe the lockdown were also fined. Bike-riders were not allowed to carry more than one person. All these laws were initiated by local youths, and they monitored daily that people followed the rules.

Young people also developed a burial strategy that eliminated the community members’ resistance to government-led burial teams. Thirty percent of the participants mentioned that many locals felt their loved ones were buried unrespectfully, given the strict measures to handle Ebola-infected corpses. Young people, who led the burial teams, therefore suggested engaging the community in the burial process by allowing them to dig graves for their loved ones and involve religious leaders.

The community here believe in praying during burial, so we trained a Christian and a Muslim leader who would work with us and pray for the deceased.

As a result, some interviewees observed that people felt their culture was respected and found the closure they needed while mourning their loved ones. Besides, the measure reduced attacks on the burial teams and promoted safe processes.

Lastly, ten percent of the youths noted that the most outstanding achievement for engaging local youths during the Ebola response was challenging people’s stereotypes of young people as being lazy. They further asserted that the local chiefs finally recognised that young people could have positive change.

#### Influence of local youth’s engagement on local development

When asked how their engagement in the Ebola response influenced their roles in their community, a few youths acknowledged that their participation was a great learning experience. Forty percent of the participant reported that the lessons learned from the Ebola experience prepared them for their careers in humanitarian work. Another interviewee also asserted that they learned community approach methods essential for their work as a youth advocate. A team leader also remarked that they learned teamwork skills and got the opportunity to demonstrate youth leadership. Working in a cross-cultural team with international humanitarian staff, according to another person, helped improve his communication skills. They further emphasised that they learned a lot from the external experts who came to fight Ebola.

Connected to the previous points, most interviewees admitted that participating in the Ebola response stimulated their passion for community service. The motivation was due to the new things they learned, the impact they created, while for others, it was the need to challenge the perceptions that people had about young people.

Due to the experience that I had with my coworkers at that time, I became more passionate to continue being a child advocate. It encouraged me to continue what I am doing, and that made me choose to become a feminist. I hope to study gender and development studies when I enrol in the university. I think if we all have the same mentality and passion, we can ignite change*.*

It also emerged that participation in the humanitarian response provided a platform for young people to engage in development issues. Some interviewees detailed that some youth who fought Ebola became ambassadors of crucial development projects after the response. One of the interviewees got global recognitions, represented youth, and contributed during the global humanitarian summit of 2016. Additionally, some youths confirmed that they initiated groups to write letters to various organisations to seek volunteer opportunities for young people in their communities. Through these roles, they said, young people can gain work experience and skills to prepare them for paid careers after school.

### Barriers that local youths faced when engaging in humanitarian response

#### Qualification for roles

Sixty percent of the youths highlighted that some humanitarian actors perceived them as unqualified due to their education levels. One respondent described as follows:

I can remember when I started working during the response, I was sitting among technical experts; by then, I did not have a degree. So they were kind of gossiping about me. They were saying they are being devalued because they were put with a young boy*.*

Yet, as another person contended, *‘*some of the staff in those organisations qualify on paper but do not have the local experience that youths have’.

On the flip side, forty percent of the interviewees admitted that not many formalities or criteria were checked for employment during the humanitarian response, like sending out adverts for jobs. NGOs partnered with local chiefs and youth leaders to identify potential youth volunteers to work with humanitarian actors. Contrarily, when Ebola cases started reducing, and some NGOs had acquired funds, some interviewees observed that many young people were dismissed from their roles as unqualified. Hence, NGOs and the government used professional qualifications to eliminate young people from specific positions according to the youths.

During the response, they engage youths to do the dirty work, but when it comes to recovery, when they started to receive some funds, they would tell you that this position is for somebody with a first degree or someone with a master's degree and you should have ten years experience. How do you expect a young man to have ten years’ experience?

#### Age discrimination

All the participants in this study reported having faced age discrimination from their colleagues and community members during the emergency response. Seventy percent of the interviewees felt that they were treated differently by their teammates in their work because of their age and lack of work experience. One youth said:

The staff always wanted me to stay in the vehicle, not letting me speak to the community members. So honestly, for my experience, it was not easy for me.

At the same time, the interviewees who did sensitisation reported that certain community members would not allow them to talk during mobilisation meetings. Young people who lead their work teams mentioned that their peers also ignored their ideas because they perceived them as too young to lead. Apart from age, a female respondent noted she faced more discrimination because of her gender, with some colleagues expecting romantic favours during the emergency response.

Due to the marginalisation discussed above, the young Sierra Leoneans widely expressed that they needed to prove their abilities. Some youths perceived that everybody wanted to see if they were capable of doing the job as a young person, while others reported that they were strictly monitored daily. According to most of the respondents, these perceptions caused them pressure and stress at work. Only ten percent contended that they did not need to prove their  expertise during the Ebola response because their roles were temporal.

Contrastingly, twenty percent of the respondents reported cordial relationships with senior colleagues who gave them the mentorship they needed to perform effectively in their roles. Interestingly, these interviewees were both team leaders and had volunteered with the organisations before the Ebola outbreak. One of them affirmed that ‘I was not treated differently because of my age. I was heading the department, so I made decisions’. On this account, it can be derived that the positions that young people held and their prior engagement with humanitarian organisations influenced the work relations with senior humanitarian experts.

#### Engagement in decision making

Exclusion from decision-making was a prominent theme from all the interviews. The young Sierra Leoneans maintained that they were not consulted by the government or organisations that engaged them during crucial decision-making. Instead, the senior staff members planned activities and instructed local youths on the day’s activities. One youth who distributed food to affected populations testified as follows:

When it comes to decisions making, well, I think that is above my level. All I know is that when food arrived, I was called upon to package it, and we will be told where we take it. You don't make suggestions, like the kind of food they give people, the kind of package.

Another person who led community outreach activities emphasised that *‘*there was not much open-door policy, you would just be ordered to do things, and you have no will to ask questions’.

While twenty percent of interviewees acknowledged that their organisations planned with young people, they revealed that the level of consultation was partial. The senior staff sought their opinions but did not implement their ideas in most cases—instead, the seniors finalised ideas in their absence without a proper feedback channel.

Some people revealed that when discussing issues, the decision-makers preferred the opinions of stakeholders’ with more prominent positions who were deemed as more experienced that young people. As observed by the youth, these stakeholders would sometimes suggest wrong ideas, but their positions of influence earned them acceptance. This description from one of the respondents explains it as follows:

The last time, we went for a meeting looking at the issue of teenage pregnancy. Then I said, “if we are looking at the issue of teenage pregnancies, why don't we involve young people themselves and involve them in sensitising their colleagues. And then people became annoyed that I was going against what the first lady of the district had said. I was like, “what is going on? I did not say anything wrong”. Everybody was giving it back to me and telling me that how could I disrespect the first lady. So I was very devastated. I felt ashamed that very moment, and after the lunch hour, I got pissed and just left the meeting.

Such instances as exemplified above discouraged young people from sharing their opinions, with many participants resonating to following instructions during the Ebola response. However, they widely expressed discontent with their marginalisation from decision making. All the interviewees strongly felt that they should have been involved in decision making as frontline workers; they understood the challenges local people faced and could recommend practical resolutions. Further, being part of decision-making processes would enhance inclusivity and affirm local youths’ self-worth as key actors in crisis response.

I should be part of the planning so that I will be able to give my own input on what is needed. Even if they are not taking it into consideration, but making my input can best satisfy me because I know I have been included.

Due to the lack of their input, some young people felt that senior staff, who mostly stayed in the office, did not care about their welfare as volunteers working in the field. As a result, others felt discouraged and chose to quit their roles.

All they care about is for us to distribute food. So I was not happy with that. So I did not work there for a long time, and then I resigned.

In a positive light, one youth advocated for his right to participating in decision making using the organisation’s established laws. The interviewee, a child during the Ebola response, reported that he did his research on the UN charter for children’s rights and used it to challenge the team. The youth said*, ‘*I had to go and do my research about the charter and use it as a tool to be involved’. Hence, his experience exemplifies how young people can hold organisations accountable and create a platform to be involved in critical processes during crises.

#### Job ranking and compensation

Fifty percent of the youths remarked that most youths were given roles that they perceived to be lowly-ranked and risky among the Ebola response teams. These included roles like being security guards, cleaners, drivers and other operational positions. The older NGO staff, by contrast, were assigned supervisory roles. Some interviewees felt that these rankings were based on the NGOs perception of young people as over-ambitious; thus, they tended to restrict their entry to administrative positions to preserve their organisational power.

They think that young people will overtake them in their positions. They think that if I understand their system or how they are doing everything, I will go and teach my colleagues how to overthrow them from those positions. They see young people as a threat who will interfere with their system of operating, so they tend to hinder and limit the participation of youths. In fact, discourage young people from getting to higher levels.

The above testimony suggests that humanitarian actors tended to keep local youths at lower job ranks as a way of holding them in check. Young people, in this case, were perceived as agitators that needed to be controlled; otherwise, if they were included in administrative roles, they would attempt to take over the organisations.

Some youths also raised the issue of compensation in their discussions. These youths felt that they were delivering more than most external experts during the emergency response, but the stipend they got was unmatched by what the rest of the NGO staff earned. Other youth leaders expressed that the pay was inadequate to sustain their livelihoods after the humanitarian response. Additionally, thirty percent revealed their employers started holding their salaries when Ebola cases began reducing. Perhaps at this point, the organisation felt they did not urgently need the services of the local youth volunteers. In extreme instances, according to ten percent of the interviewees, some organisations closed down without informing their volunteers nor paying their last stipends.

#### Stigma and mental health support

Even though this study did not seek to overemphasise the vulnerabilities of youth in crisis, the psychological challenges that young Sierra Leoneans faced due to their participation in the Ebola response emerged during the interviews.

All the youth reported stigma as frontline workers, especially those who worked in the burial teams and medical emergency departments. Sixty percent of the interviewees said their families abandoned them given the nature of the disease that was very infectious. One interviewee said the following:

It was unfortunate for me when I started working in the emergency department. My parents asked me why I was going to work there. They abandoned me. It is very interesting to tell you this. They abandoned me.

The majority of the participants also mentioned that they faced stigma from the community members; people refused to talk to them or even sell them goods with the perception that they would be infected. Landlords also gave the youth prompt notice to vacate their houses. Young people who were contact tracers were also viewed as ‘spies’, especially from community members who were reluctant to report their loved ones sick.

Additionally, sixty percent of the respondents expressed that they saw many corpses in a day, which caused them so much trauma and confusion. This testimony from one of the burial team leaders explains further:

It was my first time to take part in burial, so you find that in a day, you see like 20 corpses as an individual, and when you go to bed at night, there are a lot of things coming to your mind. You cannot sleep. You have nightmares.

In some cases, the local youths also witnessed colleagues dying, which caused daily fear. One person also mentioned that responding to a strange virus that they knew their health system could not handle was stressful and risky.

Even though twenty percent of the youths reported that their organisations provided counselling services, the rest revealed NGOs and the government primarily focused psychological support on people directly affected during the outbreak, including Ebola survivors and those who lost their loved ones.

Governments and NGOs do not think Ebola-affected everybody. They only focused on those directly affected.

Therefore, the stigma they faced, the mental challenges surrounding their work environment and their families’ abandonment left many youths lonely with limited emotional support as they volunteered to fight the deadly Ebola disease.

#### Engagements after the crisis

When asked about the continuity of their engagements with humanitarian organisations after the Ebola response, eighty percent  of the participants confirmed that they lost their jobs as they were only temporarily involved in the response activities. While twenty percent believed they did not have the technical skills to continue engaging with humanitarian actors, most youths felt that their dismissal from their roles after the crisis response was unfair. Fifty percent of the interviewees perceived that NGOs and the government only work with young people in emergencies when they need their services and later quickly dismiss them without any mentorship or support. Further, ten percent remarked that these institutions did not care about how youth’s careers progressed as long as they could help them achieve their organisational goals. One person gave the following personal experience:

I was a volunteer with one organisation for six months, then some people came from Freetown, and they said they do not need volunteers. So we were just sent out like that, without even preparing us where to go. Everyone was frustrated. Then a time will come when they say, “you people, come again, we need you.

Hence, according to the participants, this working method with young people was tokenistic and misused their skills. To the youth, it did not make sense for humanitarian actors to continuously establish the essentiality of youth engagement in their mandates and treat young people differently in the field.

On the flip side, thirty percent of the participants confirmed that humanitarian organisations contracted them for various roles in project management and advocacy after the Ebola response. Ten percent further revealed that the NGO supported their volunteers to attain higher education and vocational training. Some of the former colleagues, they mentioned, are now working as plumbers, welders and drivers within their communities.

### Strategies to meaningfully engage local youths in humanitarian response

Majority of the participants felt that humanitarian organisations and the government did not meet the general needs of young people during the Ebola response. Some youths reported high teenage pregnancy rates and that the young mothers were never supported to resume school. Many youths also remained jobless after the Ebola response with no sources of income.

Therefore, to achieve a youth-centred and youth-led humanitarian response, the participants for this study recommended that humanitarian actors critically engage the abilities of young people to gain practical skills in humanitarian response. They emphasised that young people are not lazy as most people perceive, but their lack of engagement is the challenge. Some youths dismissed the need for educational qualifications during humanitarian response. According to the respondents, youths had more experience in local crises response than some humanitarian experts. Besides, some youths might have missed education due to prevailing inequalities, thus excluding them from humanitarian activities only widened the gap.

The participants also widely expressed their desire to participate in decision-making and lead humanitarian response. Many interviewees indicated that the most effective way to meet locals’ needs affected, including young people, is by involving them in all the critical stages of the response.

Any organisation that works for young people must have to work with young people.

Young Sierra Leoneans also stressed the need for safeguarding while working with young people in a crisis setting. They emphasised that humanitarian organisations should not only focus on their desired results but also consider the youths’ welfare and protection at work. A female interviewee also reminded NGOs and the government to address gender and age stereotypes that young people faced and offer the necessary support. Another underlined the need for emotional support during severe crises. She said the following:

We also need total encouragement from people leading those institutions because what we face in the field is not easy.

After an emergency response, interviewees highlighted that NGOs and governments should extend support packages to local youths like school fees to resume education or funds to start businesses to local youth workers, thus empowering them to improve their livelihoods after crises. Youths also underlined the need for more knowledge and skill training to capacitate them for effective humanitarian response in the future.

To conclude, all the interviewees affirmed their passion and enthusiasm to participate in any emergency response in the future. Twenty percent of the youth mentioned that they would only work with organisations devoted to responsibly care for their welfare and safety while working. The respondents further advised their peers hoping to engage in humanitarian response to research different organisations to remain focused and avoid exploiting their skills.

## Discussion

This section discusses the findings grounded on the theoretical framework and based on the three research objectives. The analysis proves young people as capable agents in humanitarian response. Indeed, youths do not perceive themselves as vulnerable during humanitarian crises. If at all, they are widely reported to be the vital force in frontline response activities. While the intention of this paper was not to disparage the current efforts of humanitarian experts in crises, the results confirm that their categorisation of youth and power ambitions hindered the effective engagement of local youths in the crises response. As a result, most young people who participated in the Ebola response felt misunderstood, unsupported and eventually neglected after response activities.

### Role of local youths in the humanitarian response space

The findings of this study are consistent with existing research on young people’s participation and immense contribution to humanitarian response (Chauke 2020; Welch 2016; OECD 2020). While there were many motivating factors to participate, this study established that the majority of the local youths were driven by altruism, as suggested by Gul (2020). Local youths in Sierra Leone supported in contact tracing, conducting safe burials, food distribution, case management, awareness creation and offering psychosocial support during the Ebola response. Using their technological know-how (Gul 2020) and mobilisation abilities (Haynes and Tanner (2015), local youth developed a set of community-informed Ebola messages to dispel the myths that the majority of Sierra Leoneans had about Ebola (Kinsman et al. 2017). They also restored trust in the local health facilities, which Christensen et al. (2020) describes as essential in maximising outcomes by motivating communities’ active participation in emergency response.

Local youths also understood and recognised place-based cultural values in their communities while responding to Ebola, thus supporting Roepstorff’s (2020) previous research on the benefits of localising humanitarian action. Unlike external experts who Pacitto and Fiddian-Qasmiyeh (2013) assert may overlook most pressing challenges, the youths observed their community’s burial practices. Hilhorst (2018) also supports this claim that humanitarian experts’ exclusive focus on ethical practices can detach them from the locals and cause mistrust. Therefore, it is essential if humanitarian actors engage local youths who can develop response strategies aligned to sociocultural priorities and practices, driving positive change within their communities.

### Barriers that hinder effective local youths’ engagement in humanitarian response

Based on Sen’s capability approach, the results above prove that local youths already have the individual internal endowment, including the skills sets and knowledge to engage in and positively impact humanitarian response (Robeyns 2005). However, they lacked an enabling environment and support within the humanitarian system to fully convert their skills and abilities to meaningful functioning in humanitarian response. As Chiappero-Martinetti and Venkatapuram (2014) assert, a person’s capabilities entail the combined interaction of internal and external factors which significantly influence their achieved outcomes. In this instance, the external factors shaping local youth engagement during the Ebola emergency were primarily determined by humanitarian actors, including NGOs and the government, leading response activities.

The study ascertained these institutions’ construction of local youth responders significantly shaped their engagement during the Ebola response. Findings indicated that NGOs and the government engaged local youths only when Ebola cases became overwhelming, confirming McIntyre and Thusi (2003) and Gul (2020) claim that humanitarian institutions label young people as human resources to be harnessed when there is an urgent need. Further, this study ascertained that the engagements were reactive, as previously suggested by Mitchell et al. (2008). Haynes and Tanner (2015) assert that such categorisations of youth as mere labour enhance the exploitation of their skills and resources. Moreover, the high levels of unemployment potentially exposed young people to exploitation because of their disadvantage (Gul 2020).

In the same light, the results evidenced that many organisations neglected the emotional needs of local youth responders. Young people were seemingly perceived as adults with assumptions of strong emotional stability, an issue of labelling that McIntyre and Thusi (2003) had previously established as problematic. Instead, humanitarian actors focused on populations directly affected. Hilhorst (2018) once affirmed that such practice of categorising people for services during crises overlooks vulnerable population’s critical needs. Consequently, local youth responders missed the very psychological support that they needed.

However, contrary to the previous hypotheses, this research revealed that some international organisations perceived young people as key actors in humanitarian response. These institutions provided adequate training and safety materials to youths to work as Haynes and Tanner (2015) advocate. Further, the study indicated that these NGOs used their social negotiation skills and networks to link local youths with key community leaders for mobilising people. This proved Spiegel’s (2017) support of international organisation’s resourcefulness suitable for complex emergencies like Ebola. However, it was not clear from this study whether this perception of international organisations was influenced by the western discourse of ‘saviour’ as some youths had remarked.

At the same time, this study confirmed that some organisations used their power to construct youths as lacking in experience to qualify for specific roles, in consistent with Barnett and Walker (2015) and Bah (2013) research. In addition, young people felt that they were kept from senior administrative roles due to mistrust that they could purportedly take over the humanitarian system. Hence, humanitarian actors categorised young people as ‘troublemakers’ (Rahman 2014) and Urdal 2004) to prevent them from accessing leadership. This was a strategy for the ‘humanitarian club’ to preserve their power, as Barnett and Walker (2015) and Hilhorst (2018) had asserted.

In the same manner, humanitarian actors largely excluded young people from decision making during the response. As Ruiz-Casares et al. (2017), Tanner and Haynes (2009) point out, giving young people the right to participate in humanitarian action does not necessarily give them the actual possibility to do so effectively, especially if they lack access to decision-making processes. Therefore, as Haynes and Tanner (2015) assert, the feelings of exclusion caused discouragement among many youths, with some eventually resigning from their roles. Overall, excluding local youth responders from decision making threatened their safety as frontline workers and ignored a valuable resource for insightful and practical response activities.

According to Hart’s (1992) ladder of participation, this study ranks the degree of local youth participation during the Ebola response on the two low ranks: decorative and tokenistic. This study cannot conclude that the engagement was manipulative as local youths voluntarily joined the Ebola response to protect their communities, partly aware of the risk of fighting the infectious disease. However, the findings confirmed that humanitarian actors did not fully engage young people in designing response activities and implementation plans. They primarily assigned youths’ field responsibilities with little or no choice to share their ideas. Even though some youths indicated that they were invited to decision-making meetings and allowed to share their thoughts, they were not informed about the outcomes. Besides, senior staff ideas were preferred. This form of participation was mainly to showcase youth representativeness during meetings but with no significant impacts. Overall, the results conclude that there was no valid participation of local youths during the Ebola response.

After the humanitarian response, little attention was focused on the youths who joined the frontline to fight Ebola. Even though an influx of opportunities emerged for local youths with relief efforts, the findings showed that these opportunities were not sustainable in many cases. As a result, many local youths who participated in the Ebola response had not found long-term jobs and remained unemployed. They also repeatedly voiced their concern for thousands of idle young people. According to Ruiz-Casares et al. (2017), lack of long-term engagement causes feelings of neglect among young people and discouragement to participate in similar initiatives in the future. Therefore, organisations need to establish where local youth’s responsibilities start and end and what that means for the added value for young people as frontline humanitarian partners.

This study verified that young people’s participation in humanitarian response was an entry point for local youths to gain respect, confidence and knowledge about the wider community. The experience increased their awareness and understanding of critical development issues in their communities and built their confidence to question community members and decision-makers (Ruiz-Casares et al. 2017; Haynes and Tanner 2015). Youths also gained essential personal and professional skills and built networks for their future career development. Involving young people also created a sense of belonging (Mcintyre and Thusi 2003) that stimulated their passion for participating in local development within their communities (Tanner and Haynes 2009). Contrarily, their exclusion can cause fear, anxiety and reluctance to share their experiences in the future (Haynes and Tanner 2015).

### Strategies to meaningfully engage local youths in humanitarian response

The findings emphasise strengthening local youths’ participation and leadership at all levels in a crises response to advance inclusive and effective localisation in humanitarian action to ensure meaningful engagement of local youths in crisis response. Maximising the role that young people play in response requires supporting them to design action plans and leading their implementation, inconsistent with Haynes and Tanner’s (2015) and Ruiz-Casares et al. (2017) research. Besides, giving young people an active role in decision making equips them with experiences that they can use to build back better societies (Tanner and Haynes 2009). Additionally, the current generation of young people is educated, enlightened and more vocal. Hence, key players in the field should provide them with resources for holding humanitarian actors accountable.

The results also affirmed the need for protection as an urgent priority. As Spiegel (2017) had previously noted, the centrality of protection demands that the policies and interventions adopted in humanitarian response must address the rights of all affected persons, including local responders. In addition, participants widely expressed the need for adequate training, mentorship support and remuneration during response activities. Most importantly, the findings emphasised humanitarian actors’ obligation to promote sustainable engagement with local youths in humanitarian action or extend financial and skill training during recovery to sustain their livelihoods.

## Conclusion

Even though previous research has widely evidenced that young people are significantly shaping humanitarian response, the lack of meaningful engagement due to opposing power dynamics and labelling by key humanitarian actors motivated this research.

This study has shown that young people creatively innovatively designed strategies to tackle some of the most pressing challenges during the pandemic. Participating in the crisis response also built young people’s confidence and skills and increased their awareness and passion for local development. However, the study has also revealed that humanitarian actors did not meaningfully engage local youths in response activities as the engagements were largely reactive. Local youths faced barriers like the lack of access to decision making, age discrimination at work, lack of access to administrative roles, poor compensation, lack of emotional support and neglect after the Ebola response. The UN security council resolution rightfully states that this ‘lack of leadership, protection and support for young people exposes youth to undesirable forms of engagement’ (UNV 2018, p 1). Therefore, this research recommended strengthening and formalising youth leadership at all stages of emergency response, harnessing their protection and offering emotional support at work. There is also a need to promote long-term engagement and support to young people after crises. Such actions would ensure the effective integration of young people in humanitarian action to achieve sustainable recovery from long-term emergencies and enhance local development.

In summation, the learning from this paper cemented local youth engagement as a core component of emergency response. Even though there is adequate research on the power dynamics of different actors during emergency response, no study specifically looks into the impacts of such influences on local youths who are often the first responders during crises. Hence, the experiences of local youths during the Ebola response in Sierra Leone can act as a window into more significant questions of engaging young people in crisis response. Therefore, considering the critical issues raised in this study will enhance humanitarian action that is centred on affected populations, including young people, and promote their involvement as crucial partners for sustainable development. Future research needs to focus on a nuanced understanding of the role of local youths in different humanitarian contexts to make the research more useful and better for future practices.

## Data Availability

Not applicable.
